# Tourniquet-Related Iatrogenic Femoral Nerve Palsy after Knee Surgery: Case Report and Review of the Literature

**DOI:** 10.1155/2013/368290

**Published:** 2013-11-26

**Authors:** Juan Mingo-Robinet, Carlos Castañeda-Cabrero, Vicente Alvarez, José Miguel León Alonso-Cortés, Eva Monge-Casares

**Affiliations:** ^1^Department of Orthopedics and Traumatology, Complejo Hospitalario de Palencia, 34005 Palencia, Spain; ^2^Department of Clinical Neurophysiology, Complejo Hospitalario de Palencia, 34005 Palencia, Spain; ^3^Department of Physical Medicine and Rehabilitation, Complejo Hospitalario de Palencia, 34005 Palencia, Spain; ^4^Emergency Department, Complejo Hospitalario de Palencia, 34005 Palencia, Spain

## Abstract

*Purpose*. Tourniquet-induced nerve injuries have been reported in the literature, but even if electromyography abnormalities in knee surgery are frequent, only two cases of permanent femoral nerve palsies have been reported, both after prolonged tourniquet time. We report a case of tourniquet-related permanent femoral nerve palsy after knee surgery. *Case Report*. We report a case of a 58-year-old woman who underwent surgical treatment of a patella fracture. Tourniquet was inflated to 310 mmHg for 45 minutes. After surgery, patient complained about paralysis of the quadriceps femoris with inability to extend the knee. Electromyography and nerve conduction study showed a severe axonal neuropathy of the left femoral nerve, without clinical remission after several months. *Discussion*. Even if complications are not rare, safe duration and pressure for tourniquet use remain a controversy. Nevertheless, subtle clinical lesions of the femoral nerve or even subclinical lesions only detectable by nerve conduction and EMG activity are frequent, so persistent neurologic dysfunction, even if rare, may be an underreported complication of tourniquet application. Elderly persons with muscle atrophy and flaccid, loose skin might be in risk for iatrogenic nerve injury secondary to tourniquet.

## 1. Introduction

Pneumatic surgical tourniquets are widely used in orthopedic and plastic surgery, as well as in intravenous regional anesthesia. They enable surgeons to work in a bloodless operative field in the distal extremity. However, even if proper inflation pressures and tourniquet times are controlled, all tourniquets can cause a various range of complications, ranging from minor to fatal [1].

Local complications caused by the high pressures under the tourniquet include skin abrasions, vascular injury, postoperative swelling, loss of muscle strength, and nerve injuries [2].

In the pathophysiology of nerve injury, both mechanical compression and neural ischemia are involved [3]. Mechanical compression of the nerve causes microvascular congestion, inadequate tissue perfusion, and axonal degeneration [4], leading more frequently to a transient loss of function but sometimes to irreversible damage and paralysis.

Nerve injuries associated with tourniquet use have been reported in the literature [3, 5–10], but, even if there are studies demonstrating a high rate of femoral nerve electromyographic abnormalities after tourniquet use in knee surgery [11, 12], only few articles report permanent femoral nerve injuries [8, 13]. We report a case of tourniquet-related permanent femoral nerve palsy after patella fracture surgery without high tourniquet pressures or prolonged tourniquet time.

## 2. Case Description

Our patient is a 58-year-old woman who suffered a displaced patella fracture for which surgery was indicated. Surgery was performed on April 2011, under spinal anesthesia. The patient was positioned supine and a thigh tourniquet was placed on the left limb, which was exsanguinated with use of an Esmarch bandage, and the tourniquet was inflated to 310 mm Hg (150 mm Hg above systolic arterial pressure of the patient prior to the inflation of the tourniquet).

After preoperative skin preparation with povidone-iodine and draping in a sterile fashion, a midline longitudinal incision was performed. Fracture was reduced and fixed with two Kirschner wires and a wire in figure of eight configurations. Tourniquet was released after 45 minutes, and standard closure was performed. In the postoperative care, the patient was immobilized for 2 weeks for deambulation, allowing passive flexion and extension. After 3 weeks, crutches are allowed to be discontinued, but the patient referred inability to extend the knee. Exploration showed a moderate atrophy of the quadriceps, with paralysis of the quadriceps femoris (power 0/5).

The patient initiated kinesitherapy for empowerment quadriceps, hamstrings, triceps, adductor, and tensor fascia lata; massage therapy for mobilization and peripatellar analgesia; and Transcutaneous Electric Neuromuscular Stimulation.

Three months later, due to persistent motor deficit and the apparent atrophy of the quadriceps, a first neurophysiological study was requested. The assessments were carried out using Keypad 10 (Micromed) and Medelec Synergy (Oxford Instruments) as electromyography (EMG) equipment. Nerve conduction studies were performed following standard protocols. Nerves explored included both left and right femoral nerve as well as common peroneal nerve, posterior tibial and sural in the left lower limb. Electromyographic study was performed in the left vastus lateralis and medialis, adductor longus, tibialis anterior, and L3-L4 lumbar paraspinal muscles.

In the first study, the left femoral nerve conduction was preserved, as well as the other peripheral nerves. However, EMG study showed a quadriceps injury in the form of low voluntary muscle activity and a loss of motor units with maximal effort. No significant anomalies were revealed in the rest of muscles explored. These data were interpreted as a severe axonal neuropathy of the left femoral nerve.In order to exclude other aetiology that could have caused the femoral nerve palsy, the patient underwent magnetic resonance angiography, which allowed us to rule out compressive hematoma, angiomas, or direct muscle damage (not suspected clinically).

**Figure 1:**
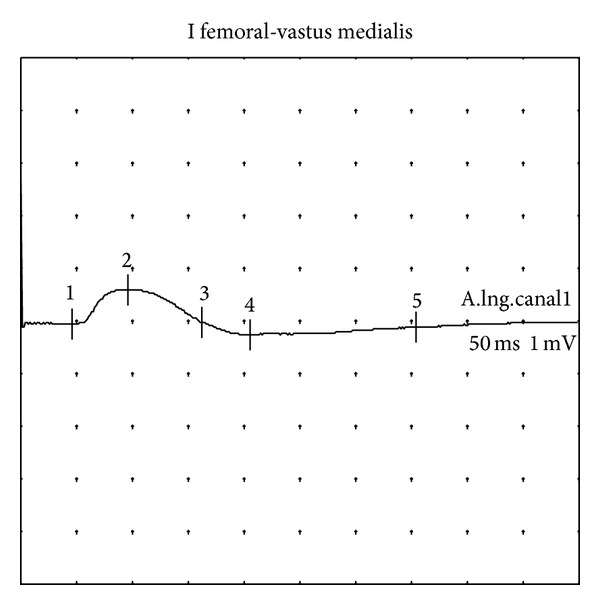
Left femoral nerve. Compound muscle action potential showing a marked amplitude reduction (0.7 mV). Study 2 (9 months).

**Figure 2:**
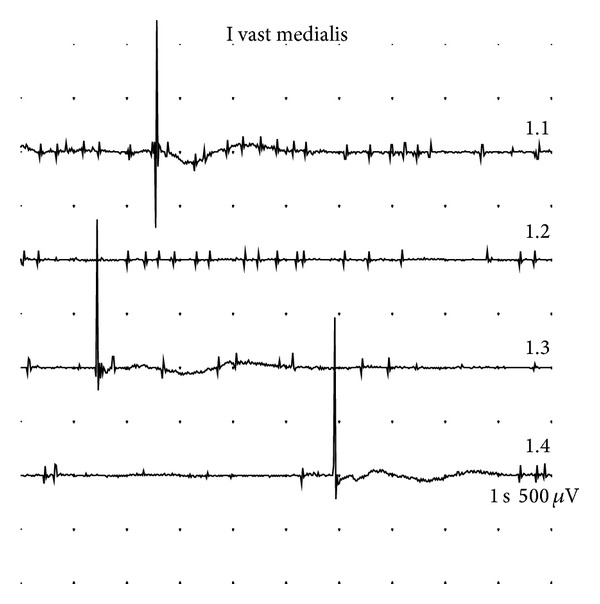
Left vastus medialis. Pathological spontaneous activity (fibrillation potentials, fasciculations). Study 2 (9 months).

Nine months after surgery, hardware is removed. In the absence of clinical improvement, it was decided to perform a second study of control, nine months after the first one, which showed a worsening of the parameters evaluated, with greater loss of nerve fibers in the left femoral nerve conduction and profuse presence of pathological spontaneous activity, expression of active axonal damage, or an underlying progression (Figures 1 and 2). The fall of motor units recruited in the pattern of maximal effort in quadriceps was still very clear and significant, although it should be noted that the muscle was not completely denervated.

**Figure 3:**
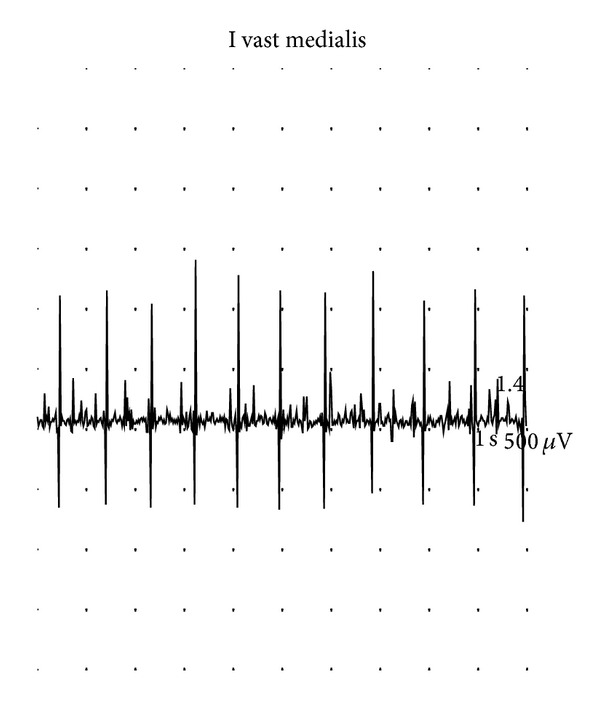
Vastus medialis. Pattern at full effort reveals significant loss of motor units. Study 3 (18 months).

**Figure 4:**
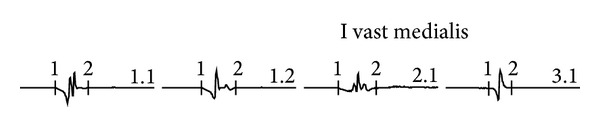
Vastus medialis. Motor unit action potentials highly polyphasic, low voltage, and short duration. Study 3 (18 months).

**Figure 5:**
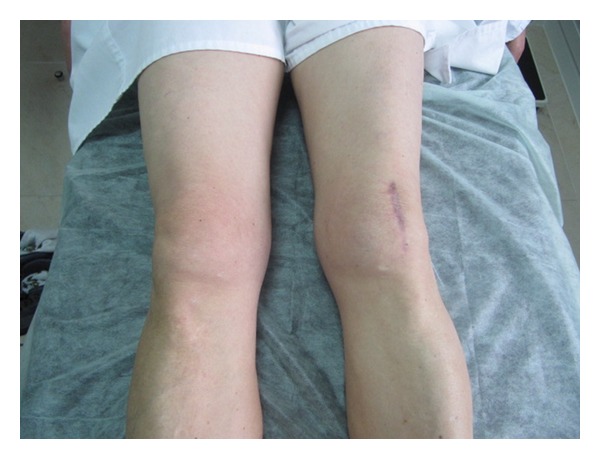
Muscle atrophy after 18 months.

Finally, eighteen months after the surgery, without clinical changes, a new EMG study was practiced, which showed a nearly complete disappearance of pathological spontaneous activity and stabilization in neurogenic pattern (Figures 3, 4 and 5). The features of the very limited measurable motor unit action potentials action were compatible with a minimal, incipient, and obviously insufficient reinnervation. The femoral nerve conduction revealed no significant changes.

## 3. Discussion

Iatrogenic femoral nerve injuries may be related to abdominal and pelvic surgery, inguinal hernia repair, hip surgery, regional anaesthesia [14], but, to our knowledge, only two cases of iatrogenic tourniquet-related femoral palsy in knee surgery have been reported in the literature [8, 13, 14].

In normal conditions, when using pneumatic tourniquet following standard recommendations, the incidence of major neurological sequelae is extremely low, being estimated to one per 6155 in the arm and one in 3752 in the lower limb, with an overall incidence of permanent injury of 0.032% [15]. Nevertheless, the appearance of subtle lesions in clinical examinations, or even subclinical lesions only detectable by nerve conduction and EMG activity [11, 12] is relative frequent.

In our patient, femoral nerve injury was attributed to the compression of the tourniquet, as other causes of iatrogenic femoral nerve injury were excluded, as direct injury or compression by hematoma or pseudoaneurysm, or palsy secondary to regional anaesthesia (femoral nerve block), as a spinal anaesthesia was performed. Electromyographic studies also excluded iatrogenic injury of L3-L4 spinal roots.

Middleton and Varian [13] report one single case of femoral and sciatic nerve palsy after four and a half hours of tourniquet use. Due to prolonged use of the tourniquet, this nerve injury would not be rare in accordance with Horlocker et al. [3], who have found a strong correlation of nerve injury with prolonged total tourniquet time with an approximate threefold increase in risk of neurological complications for each 30 min increase in tourniquet inflation. This correlation is not supported by Odinsson and Finsen [15], as, on the basis of a questionnaire in Norway, most of the nerve complications occurred after the tourniquet had been inflated for less than two hours at a pressure of 300 mm Hg or less.

Kornbluth et al. [8] report the case of one patient who sustained a complex arthroscopic ligament reconstruction. This patient sustained a long surgery in which tourniquet was inflated and deflated for four times, with variable intervals without pression. In total, tourniquet was inflated to a pressure of 300 mm Hg for 280 minutes. The deleterious effects of increased tourniquet duration and inflation pressure on the frequency and severity of neuropraxia have been demonstrated in laboratory and clinical investigations [12, 16, 17], but the efficacy of tourniquet release for reperfusion intervals in decreasing ischemic injury to nerve is unclear. Horlocker et al. [3] also found that a tourniquet deflation (reperfusion) interval only modestly decreased the risk of nerve ischemia.

Peripheral neuropathies associated with the use of pneumatic tourniquet have a double pathophysiologic mechanism: compression and ischemia. Compression plays a more important role in the 2-3 first hours; after that the deficit of blood flow can cause permanent structural changes [18]. Nerve injury directly caused by compressive effect is mainly localized under the cuff [18], where the greatest local distortion is produced, and from this site tends to progress towards distal territories. Ischemia, however, although it affects the nerve as well, has a greater impact on muscle; in the early stages it is usually limited to the occluded area, while after four hours of tourniquet use its effects spread distally [1]. In this case report, considering the brief duration of tourniquet use (less than one hour), we think that only the first mechanism was involved in the development of sequelae.

Because tourniquet-related neural injury has been linked to mechanical rather than ischemic factors, mechanical stress merits the most focus for preventing nerve injury [19]. Tourniquet should be applied to the proximal part of the limb at the greatest circumference because the muscle bulk at that site is the greatest, and hence it affords a greater protection against nerve injury [20], and so, elderly persons with muscle atrophy and flaccid, loose skin might have a higher risk of nerve injury [21].

## 4. Conclusion

Even if complications are not rare, safe pressure for tourniquet use remains a controversy, as well as the real correlation between total tourniquet time and risk of neurological complications. Nevertheless, subtle clinical lesions of the femoral nerve or even subclinical lesions only detectable by nerve conduction and EMG activity are frequent and might often be underdiagnosed. Based on the literature review, permanent tourniquet-related nerve palsies are rare but may also be under reported.
